# Supplementary material for: Content-rich biological network constructed by mining PubMed abstracts
Source: BMC Bioinformatics. 2004 Oct 8;5:147. doi: 10.1186/1471-2105-5-147 (PMC528731; doi:10.1186/1471-2105-5-147)
Supplement: Additional File 2 — The original results of the above study (non-essential files are deleted to keep the file size under the limit set by BMC bioinformatics). [file 1471-2105-5-147-S2.bz2 › chilibotAdditionalFile2/dip05/727ID9822594E18076/html/HLYB_HLYA.html]

 


 **HLYB** and **HLYA** 
  
Found 91 abstracts in PubMed, retrieved 05.  
 

 What does Google say? 
 PDF only 
| .edu only 

---

**Interactive relationship** (e.g. stimulation, inhibition, etc)

**Neutral relationship**- The mutant  **HlyB**  HlyDDelta45 inner membrane complex engaged the hemolysin  [ **HLYA** ]  substrate, but this substrate engaged complex failed to trigger recruitment of TolC.  Ref: 11676535 J Mol Biol, 2001

**Non-interactive relationship** (e.g. studied together, co-existance, homology, etc.)

- The structure and function of the components of the  **HlyA**  secretion apparatus,  **HlyB** , HlyD and TolC, have been studied in great detail.  Ref: 11755084 Trends Microbiol, 2002
- The presence of fragments of genomes  **hlyA** ,  **hlyB** , papAH, papC, sfaG, sfaA and kps MT, associated with the pathogenicity islands of Escherichia coli, in clinical strains of other genera of the family Enterobacteriaceae, has been experimentally evaluated with the use of PCR.  Ref: 12506618 Zh Mikrobiol Epidemiol Immunobiol< MedlineTA>Zh Mikrobiol Epidemiol Immunobiol, 2002
- Taking these results together, we propose a model for an early stage of initiation of secretion in vivo, in which the NBD of  **HlyB** , specifically recognizes the C terminus of the transport substrate,  **HlyA** , and where secretion is initiated by subsequent displacement of  **HlyA**  from  **HlyB**  by ATP.  Ref: 12662939 J Mol Biol, 2003
- We have purified the nucleotide binding domain of  **HlyB**   **HlyB**  NBD and a C terminal 23kDa fragment of  **HlyA**  plus the His tag HlyA1, which contains the secretion sequence.  Ref: 12662939 J Mol Biol, 2003
